# The Impact and Wider Implications of Remote Consultations for General Practice in Norway: Qualitative Study Among Norwegian Contract General Practitioners

**DOI:** 10.2196/63068

**Published:** 2024-12-17

**Authors:** Børge Lønnebakke Norberg, Bjarne Austad, Eli Kristiansen, Paolo Zanaboni, Linn Okkenhaug Getz

**Affiliations:** 1 General Practice Research Unit Department of Public Health and Nursing Norwegian University of Science and Technology Trondheim Norway; 2 Norwegian Center for E-health Research University Hospital of North Norway Tromsø Norway

**Keywords:** remote consultations, e-health, digital medicine, telemedicine, impact, downsides, disadvantages, pitfalls, safety, general practice, family medicine, practice organization, ecology of healthcare, remote consultation, monitoring, teleconsultation, social determinants of health

## Abstract

**Background:**

The digital shift toward remote consultations in general practice needs ongoing monitoring to understand its impact on general practice organizations and the wider health care system.

**Objective:**

This study aimed to explore how remote consultations impact on contracted general practitioner (GP) practices and how GPs perceive the implications of this uptake for the overall health care system.

**Methods:**

In total, 5 focus groups were conducted with a total of 18 GPs from all 4 health regions of Norway in 2022. The material was subjected to Braun and Clarke’s thematic analysis.

**Results:**

The analysis yielded six themes: (1) the design of novel effective clinical pathways: remote consultations empower GPs to tailor new effective clinical trajectories, blending modalities to address diverse needs across clinical episodes—from initial triage, through investigations to case closure; (2) increased workday flexibility: remote consultations introduce variability into daily work, allowing GPs to adjust patient contact intensity, and leading to a less stressful work-home balance; (3) erosion of organizational boundaries: easy remote access to GPs appears to reduce patients’ tolerance for minor illness and self-care, hindering effective gatekeeping and shifting GPs’ focus from proactive to more reactive work, increasing work-related stress; (4) degradation of clinical shrewdness: confronted with an increasing amount of unsorted and trivial remote inquiries, GPs observe challenges in detecting and prioritizing serious cases; (5) dilemmas related to responsibility, ethics, and legislation: remote consultations highlight a tension for contract GPs between legal responsibilities and ethical obligations, with implications for patients with limited health literacy; this may entail suboptimal evaluation or delayed treatment—potentially contributing to increased health care inequity; and (6) retaining clinical core values in a changing world. Overall, GPs affirm that remote consultations have come to stay and describe efforts to effectively manage the advantages and disadvantages inherent in such interactions to safeguard clinical effectiveness and organizational sustainability of primary health care.

**Conclusions:**

The widespread adoption of remote consultations in the Norwegian contract GP scheme fundamentally reshapes the dynamics of GP work and the overall health care system. Awareness and proactive management of these changes are essential for maintaining sustainable, high-quality primary health care.

## Introduction

The COVID-19 pandemic catalyzed a rapid digital shift in the way consultations are provided to patients in general practice around the world [1-4]. The conventional practice of physically visiting a general practitioner (GP) has undergone transformations with the advent of remote modalities, including video, text-based, and telephone consultations [5-10] (Textbox 1).

**Facts about remote consultations and the contract general practitioner scheme in Norway.**

**Remote consultations**

This study examines 3 types of remote consultations—video, telephone, and text-based. Video and telephone consultations are real-time and synchronous, conducted through phones, tablets, or computers. In contrast, text-based consultations are asynchronous, written exchanges between the patient and doctor. Typically, text consultations start as the patient submits a free-text request, sometimes accompanied by a predefined questionnaire.

During the COVID-19 lockdown in Norway, remote consultations surged, accounting for nearly two-thirds of all general practitioner (GP) consultations. As of 2024, they have stabilized at 20%-25% of all consultations [11]. Video consultations, however, remain relatively uncommon [12], while telephone and text consultations continue to be widely used, partly explainable by the reimbursement system [13]. Video consultation technology is increasingly integrated with patient record systems [13]. Use of remote consultations is not mandatory for GPs; each GP decides whether to offer text, video, or telephone consultations, and a small percentage opt out of 1 or more of these options.

In Norway, 80% of patients use a national digital health portal, Helsenorge, for booking appointments, prescription renewals, and text consultations with the GP [14].

In general, patients choose the consultation method [11]. Telephone and video appointments are often triaged but usually scheduled for the same day. Non-urgent face-to-face consultations usually have a waiting period of 2 days to 3 weeks [11,14]. GPs are required to respond to text consultations within 5 working days, but many respond sooner. As a result, remote consultations generally offer quicker access compared with physical appointments [11].

**The Norwegian Contract General Practitioner Scheme**

Norway’s health care system is founded on the principles of universal access, decentralization, and continuity of care. Since 2001, all Norwegian citizens have had the option to enroll with a GP or change their enrollment—a choice exercised by 99% of the population [15]. This system is designed to ensure continuity of care, with GPs acting as coordinators of municipal services and gatekeepers to specialized care. Contract GPs typically work in groups of 3-8 doctors, supported by health secretaries and occasional nurses. As of 2024, Norway has 5391 contract GPs, each responsible for a defined patient list, typically consisting about 1000 citizens. GP centers are open from 8 AM to 4 PM on weekdays and GPs often take part in evening and night emergency shifts in their municipality or region [15].

A contract GP in Norway typically manages clinical episodes of care [16] through a sequential approach in multiple steps. This process may include triage, history taking, clinical examination, a period of observation, supplementary tests (like blood tests or imaging), specialist referrals, result review, treatment planning, follow-up, and episode closure.

The income system for contract GPs varies. Approximately 20% are on fixed salaries. The remaining 80% operate on a semiprivate basis under regulated contract agreements [15]. They receive a fixed annual payment per patient to help cover practice expenses. In addition, they are reimbursed per consultation, with extra fees for specific procedures such as electrocardiogram tests, minor surgeries, and talking therapy. GPs also charge out-of-pocket fees from patients, typically around 230 NOK (US $21) per consultation (whether physical, text, telephone, or video) up to an annual cap of 3165 NOK (US $286) per citizen (for essential medical expenses), and costs beyond this cap are generally covered [17].

In addition to the well-established public GP scheme, a few fully private providers offer medical services at the patient’s expense. This includes private digital health companies that offer consultations exclusively through video or text. While private health insurance in Norway remains limited, it is gradually expanding. Due to the well-reputed contract-GP system and a long-standing GP shortage, GPs generally have minimal concern about losing patients to private competitors for purely financial reasons [11,14,15].

This shift brings forth numerous opportunities and challenges in both clinical and organizational realms. Consensus remains elusive regarding the appropriateness of remote consultations for specified reasons for contact [7-9]. Existing research, partly predating the COVID-19 era, points to context-related variations in the suitability of remote consultations [7-9,18,19], for instance, where the consultation in question fits within a clinical episode—understood as a consecutive, thematically related series of clinical contacts [16]. Some studies have highlighted the higher suitability of remote consultations when the patient and presented health issue are known beforehand [5,7,20-22]. In many instances, face-to-face consultations are seen as the preferred option when feasible [22,23].

GPs acknowledge that remote consultations have a multifaceted impact across the various levels of health care [24-26]. The health services can be viewed as an ecological system [27], emphasizing how changes at one level have ripple effects across the entire system. In a systems perspective, remote consultations can be discussed in relation to individual encounters (micro level), local practice organization (meso level), and the overarching health care system and a broader sociocultural context (macro level) [5,22-25].

At the consultation (micro) level, text consultations have been proven effective for several purposes such as simple inquiries, elucidating diagnostic workup plans and test results, monitoring of chronic conditions over time, and extending sick leave [18,19,28-33]. During the pandemic, GPs documented the appropriateness and safety of telephone and video consultations for triage in acute and subacute settings [34-40], with subsequent findings supporting their suitability for follow-up on chronic issues and administrative purposes [5,41,42]. Attempts to analyze relationship building in remote consultations have yielded mixed results [6-10]. Uncritical adoption of remote consultations may compromise clinical observations and cause patients to withhold crucial information from their doctors [20,21,43,44]. This could result in missing vital details and, consequently, less precise diagnoses [7,10,44,45].

At the practice organization (meso) level, the digital shift has been shown to increase the workload for both GPs and auxiliary personnel, extending working hours and generating a higher volume of requests [1,24,25], thereby heightening stress levels and limiting opportunities for teaching clinicians [9,10,24]. However, GP offices also experience greater flexibility in how patients can contact their physician, and, in some instances, there is reduced triage work for support staff [5-7,20] and generally improved efficiency [1].

At the wider system (macro) level, recent research has explored potential challenges of remote consultations on health care system dynamics, including overmedicalization and reduced opportunities for addressing social determinants of health [5,21,24,25,46,47]. The heightened accessibility of GPs through remote consultations could reduce the barrier for patient-initiated contact [24,25,44,45], raising concerns about GPs’ time allocation [20] and potentially undermining their pivotal gatekeeping role [24,48]. Uncertainty persists regarding whether remote consultations will result in disparities in access to digital services [48-53].

An essential question is how the use of remote consultations will stabilize in the “new normal” after the COVID-19 pandemic [6-10]. Since implementation was expedited by a natural experiment, understanding the changes that have occurred becomes imperative [1-4]. Unforeseen changes may have transpired [18,19,37,54]. Gaining more knowledge about the potentials and pitfalls associated with the use of remote consultations in various clinical and organizational contexts is vital.

This study is part of a larger project aimed at investigating the ramifications of remote consultations [55]. In an associated paper, we will explore the microlevel dynamics of doctor-patient communication in remote consultation. The objective of this paper is to explore how remote consultations impact on GPs’ practices (meso level) and what implications the GPs see for the health care system (macro level). Our exploration focuses on GPs’ overall experiences with remote consultations. All 3 remote modalities were discussed (Textbox 1). We did not aim to specifically disentangle the discussion into individual modalities, rather the aim of this study was to address the new practice normal in the wake of the digital shift.

## Methods

### Overview

We conducted a qualitative focus group study involving 18 Norwegian contract GPs. Focus groups were chosen as a suitable method for examining attitudes, experiences, and areas where existing knowledge is limited, allowing for the exploration of tentative and potentially conflicting viewpoints [56-60]. We strived to follow the Standards for Reporting Qualitative Research (consolidated criteria for reporting qualitative research [COREQ]) guidelines, refer to the checklist in Table S1 in Multimedia Appendix 1.

Our research team included 2 contract GPs (BLN and BA) with experience in remote consultations, a former GP who is now a full-time professor in behavioral sciences in medicine (LOG), and 2 digital health researchers (PZ and EK) with a background in engineering and economics, respectively. LOG and BA are senior members of a GP research unit, bringing an academic perspective on general practice, particularly in areas such as continuity of care, patient-centered communication, and relational trust. BLN also has experience as a lecturer in clinical communication for medical students.

### Recruitment Procedures

For the focus group discussions, we used purposive sampling to recruit experienced contract GPs who were familiar with remote consultations. Potential participants were contacted through phone and email, targeting a diverse range of geographical locations, including both urban and rural municipalities. We also aimed to represent different income models within the contract GP scheme, including fixed salary, subsidized positions, and capitation-based (paid per capita) systems.

Participants were drawn from two sources—3 focus groups were composed of GPs from preexisting postgraduate educational groups, while the other 2 consisted of individually recruited GPs. All but one of the GPs approached agreed to participate. While some participants had previous professional connections with members of the research team, there were no personal relationships that could compromise the integrity of the data. The inclusion of preexisting educational groups, where participants had established relational trust and experience in discussing complex professional matters, was viewed as a positive factor that facilitated open dialogue.

Initially, we planned to conduct 4 focus groups. However, after preliminary analysis of the first 4 sessions, a fifth group was added to enhance information power [57-60]. Following the initial contact, participants were provided with written information detailing the study’s purpose, data management practices, and withdrawal procedures (“Appendix S2” Multimedia Appendix 1). No participants were excluded or chose to withdraw from the study.

A summary of participant characteristics is presented in Table S2 in Multimedia Appendix 1.

### Data Collection

The focus group interviews were conducted between January 15 and April 20, 2022, that is, after the lifting of all pandemic-related restrictions in the Norwegian health care sector. In total, 3 sessions were held in person at local medical centers, while 2 were conducted remotely through the Microsoft Teams platform, facilitating the participation of GPs from rural areas and various other regions of Norway. All interviews were conducted in Norwegian. 

Before each recorded session, participants were reminded of the voluntary nature of their involvement and provided informed consent (Appendix S2 in Multimedia Appendix 1). They were instructed to ensure the anonymization of any patient-related information discussed during the sessions. In addition, participants submitted deidentified demographic information about themselves and their practices.

Moderators BLN and BA led the interviews using a semistructured interview guide, collaboratively developed by BLN, BA, and LOG. The guide incorporated open-ended, flexible questions (“Appendix 1” in Multimedia Appendix 1) and was informed by the research team’s previous work on remote consultations [4,20], which provided a solid foundation of physician insights that shaped the thematic focus. The guide was pilot-tested with 2 GPs and 2 nonmedical PhD students with experience in qualitative research.

Each focus group session lasted between 95 and 120 minutes, was audio-recorded, and subsequently transcribed verbatim. Participants received a reimbursement of approximately 1000 NOK (US $90), reflecting about half the typical rate for 2 hours of clinical consultation work.

### Data Analysis

Reflexive thematic analysis, as outlined by Braun and Clarke [56-58], was used to identify patterns of meaning across the data. The initial stages of analysis were conducted independently by LOG and BLN, with LOG performing manual coding and BLN using NVivo software (Lumivero) for digital processing. Throughout the process, BLN and LOG compared and discussed their findings, followed by collaborative sessions with BA. Relatively early in the analytic process, it was decided to include a fifth focus group to strengthen the information power, particularly regarding a perceived lack of emotional exchange during remote consultations.

The primary analytic approach was inductive, derived directly from the data, though it was inevitably shaped by the researchers’ professional backgrounds. Preliminary themes and potential candidates for final themes were shared with the entire research team and reviewed with input from 2 external project collaborators (refer to the Acknowledgments section). At this stage, significant revisions were made to the thematic framework. In the final phases of analysis and manuscript preparation, regular discussions among all authors helped to challenge individual understandings and assumptions, thereby strengthening the validity and relevance of our work.

### Ethical Considerations

All participants provided written informed consent before their involvement in the study. As the research was conducted with health care professionals and did not involve patients or the handling of sensitive health information, it did not fall under the purview of requiring approval from the Regional Committees for Medical and Health Research Ethics, in accordance with the Norwegian Act on Medical and Health Research §2 and §4. The data management procedures received approval from the Norwegian Center for Research Data (NSD/SIKT; reference 531672). Interview data were securely stored and managed in compliance with national and institutional data protection regulations. Throughout the transcription and publication processes, personal and demographic details were anonymized to prevent the indirect identification of participating GPs.

## Results

### Overview

In all focus groups, GPs noted that remote consultations had led them to adopt new organizational methods in their daily clinical routines (ie, at meso- and macro-organizational levels) compared with prepandemic times, indicating a permanent shift. There was general agreement that remote consultations entail both promising potentials and pitfalls, and that we are still early in defining their optimal use. Our analysis of the impact of remote consultations in contract GP practices resulted in 6 themes.

### Design of Novel Effective Clinical Pathways

A central topic was how remote consultations facilitate the design of effective clinical pathways, referring to a sequence of contacts unfolding over time throughout an episode of care (as described in Textbox 1). All GPs experienced that access to remote consultations broadens the range of options for deliberate cultivation of this well-proven approach: taking each step as needed and adjusting if necessary—a hallmark of good general practice, based on unrestricted access and continuity of care.

I find that the balance and adaptability between in-person consultations and electronic communication methods, such as phone calls and other digital platforms, work seamlessly for my needs.

ID7

Diverse ways of designing clinical trajectories were discussed in the focus groups. There was general agreement that the new remote modalities, including video and attachment of photos to text consultations, improve triage of severity across distances in acute or semiacute cases. GPs in rural areas were particularly satisfied with this advance and felt that considerable travel time and resources were saved. In this context, several respondents specifically mentioned the possibility of visually evaluating a feverish child’s overall condition and responsiveness, enhancing the GP’s ability to give targeted advice and safely prioritize.

In relation to nonacute matters where several contacts are often involved, the GPs’ opinions differed regarding “optimal” trajectory design. Some GPs preferred to start by a “remote first” consultation, simply to get a brief overview.

You can unlock the potential remotely and then schedule subsequent consultations, often physical ones requiring physical examinations or blood tests, in advance of new remote sessions.

ID2

Some GPs discussed what we in the analytic phase came to designate an “early, effortless commencement” phenomenon. Well-selected, suitable, and nonacute reasons for contact can be digitally launched rapidly and smoothly, with less total effort than before.

I experienced this as an advantage, because it has become possible to handle things that are not acutely urgent, but which must be dealt with earlier than the first available regular consultation appointment.

ID5

In contrast to this, other GPs clearly preferred a “physical first” consultation, with the potential for remote follow-up, if needed. GPs who preferred the “physical first” approach reflected further on the advantages of getting a comprehensive “feeling” for the case before advancing further. They argued that this will reduce the risk of missing relevant contextual or complicating factors and thereby increase effectiveness in a longer perspective.

I like to start with a physical consultation to sense the non-verbal clues and include the wider context.

ID16

Before the digital shift in 2020 which introduced both new remote modalities and reimbursement of remote consultations, most GPs had been accustomed to losing contact with their patients when they underwent treatment organized by other carers or institutions. Examples include patients who undergo chemotherapy for cancer or a rehabilitation program after a severe disease or accident. Such periods typically entail critical periods with a substantial risk of frustrations, complications, and back-lashes for the patient. Many GPs described how remote consultations had given possibilities to apply their knowledge of their patients as persons to follow up and encourage in such instances. A related gain with remote consultations was described as maintaining continuity of care and offering advice to patients despite temporary geographical separation for other reasons, for example, retired patients on long-term holiday and young people with established medical conditions who move away to study.

Another finding related to the design of effective clinical pathways can be referred to as “just-in-time consultations.” The GPs themselves did not explicitly use this term, but the phenomenon was described in several instances. It refers to how remote consultations facilitate contact with vulnerable or unstable patients with limited stress tolerance and recurrent risk of mental collapse—by one GP contract metaphorically referred to as my “house of cards-patients.” For these patients, a lot is at stake. High accessibility and short, remote consultations can sometimes counteract a downward spiral of anxiety and suffering, reducing the risk of emergency calls and even acute hospital admissions.

Low-threshold video consultations work well for such patients: We make an agreement allowing them to contact me via video, even if we do not have an appointment. When I notice they have made contact, I call them back.

ID17

### Increased Practice Flexibility

GPs in all groups agreed that variation during the day is important for work-related well-being. They described different aspects of this variation. For a start, regarding reasons for contact and levels of clinical complexity, a mixture of “simple” and “heavy” issues can be optimal.

I have numerous 30-minute physical consultations delving into psychiatric matters extensively. This can become quite burdensome at times. However, since they receive weekly follow-ups, strategically incorporating simpler topics in between on remote sessions, proves to be perfectly manageable.

ID7

In addition to this, GPs described how remote consultations give opportunities to vary and regulate the mental and emotional intensity of contact during the working day.

When I put on a headset and engage in phone calls, I find myself walking around the room, listening intently, stretching, and occasionally gazing out onto the pedestrian street below. For me, these phone calls serve as a brief respite in my day-to-day routine. The patients appreciate this approach, and I personally find it to be an enriching aspect of my daily routines.

ID9

Interestingly, the GPs held differing experiences regarding the degree of mental and emotional investment associated with different types of remote consultations. Some described contact on video as a significant relief in contrast to physical encounters, while some found video just as demanding.

Another reported advantage was related to safeguarding the GPs’ professional integrity in encounters with highly demanding patients, who repeatedly attempt to push the doctor’s limits. GPs felt able to make professionally solid but unpopular decisions and end discussions more effectively, with the use of remote consultations. In some instances, it could even be a question of personal safety for the GP.

I recently had a remote consultation that I was relieved was not in person. The patient showed aggressive behavior and, in the end, despite his demands for medication, I concluded that it was not appropriate. His reaction was quite intense, leaving me grateful for the distance provided by the remote setting.

ID1

A final, welcomed aspect of flexibility associated with remote consultations was improved work-from-home possibilities. The stress of having to finish the working day before the closure of the kindergarten can be diminished as most GPs have established an additional, digital working station at home.

### Erosion of Organizational Boundaries

GPs in all focus groups described how remote consultations had come to affect the entire “ecology” of their clinical practices. Overall, patient inquiries have become more frequent, and more often than before with limited medical relevance. Referring to the seminal paper “Ecology of medical care” by White et al [27], known through undergraduate or postgraduate education to numerous Norwegian GPs, participants described how patients’ ability to self-care seems to be on the decrease.

I am concerned about the trend towards overuse and medicalization where an increasing number of all patients being aware of one or another symptom, who previously might have sought advice from friends or family, now choose to contact their doctor instead of adopting a wait-and-see approach.

ID18

It appears as if the traditional “wait and see” phase between symptom debut and health contact is often abandoned.

…many symptoms currently presented to the GP are self-limiting and would have resolved themselves before ever necessitating a consultation with the doctor in a pre-digital era.

ID5

Upon further discussion in the groups, it became clear that the feeling of being overflowed by banalities pertained particularly to text-based consultations, which bypass conventional appointment triage, previously performed by health secretaries. This has clear consequences for the GP’s professional roles and responsibilities.

It is particularly noteworthy with these text-based e-consultations; they bypass any secretarial screening or triage process and land directly in my inbox. Only I see them. It is remarkable that as an individual, you have a direct line to a named person within the public healthcare system. One can only speculate on the outcomes if similar direct access were available in other sectors of the welfare system.

ID5

Feeling that even more of their daily work becomes dominated by issues of minor medical relevance, GPs noted that their position as essential, qualified professional actors in the health care frontline is under siege.

We GPs become limitless and must answer absolutely everything. It deprives people of the ability to take care of themselves, for things that they should not bother the health services for.

ID8

Once unfiltered requests reach the GP’s desk, it becomes a challenge to close cases, even seemingly trivial ones. GPs feel obliged to provide some sort of safety net, as remote consultations entail an increasing risk of missing critical issues that would most likely have caught the GP’s attention in a physical meeting. The threshold for follow-up decreases, even in trivial cases.

We automatically schedule patients for an appointment to discuss these results.

ID2

Although participants noted that remote consultations can make it easier for a GP to reject what they see as unwarranted requests as noted above, a different phenomenon was reported when it came to decision-making in “gray-zone areas” entailing uncertainty. Clinical judgment can definitely be harder to apply when you are not in the same room as the patient, and the GP might tend to “give in” due to unavoidable uncertainty. This could for instance be the case for drug prescriptions and sick-leave certificates.

I am concerned that the digital shift contributes to increased medicalization because it lowers the threshold for contacting the GP. Consequently, as a GP, one might not give as much thought before suggesting medication.

ID18

A particularly challenging phenomenon related to text consultations was eagerly discussed, referring to what a GP metaphorically called “the undetonated inbox.” As text consultations enter Norwegian GPs’ inboxes without any screening or triage, they may include serious and acute problems, although the electronic submission system informs patients that text consultations are not for urgent matters.

I have encountered patients reporting chest pain via text consultations, necessitating urgent admission to the hospital’s emergency room.

ID 11

Many GPs agreed that the unpredictability associated with the inbox has come to represent a new kind of work stress. One coping mechanism involves prioritizing control over the inbox above other pertinent tasks, not only during the workday but also extending into personal time, which can disrupt the work-home balance.

This has been the worst part of text consultations: If I do not check the inbox and come to work on Monday morning, there might be 120 messages in the inbox [including reports and prescriptions].

ID13

Before the rapid digitalization in 2020, GPs were used to having some time and space to reflect on recent consultations and plan future activities proactively. After the implementation of text consultations, several GPs noted that their practice style had become reactive and restrained.

Discussing “the undetonated inbox” phenomenon and the urge to monitor the inbox, some GPs shared advice to take more control.

To cope with this abundance of remote consultations [not seen by health secretaries] … I mostly wait a couple of days. At least with those who “need” something of minor medical importance quickly. This is an important thing for creating sustainability if you are going to become accustomed to utilizing it.

ID12

### Degradation Of Clinical Shrewdness

Associated with eroded organizational and professional boundaries, several GPs discussed a risk that they might gradually become less shrewd in their demanding role as generalist clinicians in the frontline of the health care system. In a stream of unsorted and increasingly trivial problems, their capacity to maintain focus and perform the core task of spotting serious health problems and prioritizing the right patients for further investigation and potential referral might become blunted.

I believe that as more people reach out to us, the more serious cases of illness may become diluted, resulting in fewer noticeable instances, and we might miss detecting issues when they eventually arise.

ID18

Remote consultations entail less informative encounters and, over time, more superficial relationships, the GPs explain. To function optimally, a GP needs a minimum of physical encounters with a person to be able to develop an “intuitive” ability to distinguish whether a next, vague, problem is trivial or indicative of something serious. Present in the same room, the doctor will have learned to appreciate the patient’s usual appearance and habitus as a baseline for future evaluations.

[Based on physical encounters] I have utilized it to develop an intuition, a gut feeling, over time. It is as if I have become a bloodhound for illness: I can ‘sense’ cancer. And as a result, I also find myself ‘sensing’ illness while conversing with patients over the phone and during video calls.

ID13

The dilemmas related to the maturation of clinical competence-building are also, according to the participants, relevant for the training of new GPs:

If clinical newcomers dive directly into the digital world; are doctors losing the ability to detect cancer remotely?

ID14

### Dilemmas Related to Responsibility, Ethics, and Legislation

Participants in all focus groups explored the boundary between formal directives and requirements on the one side and their personal sense of moral obligation on the other. The GPs felt that optimal quality of care is not ensured as long as patients remain free to select consultation type (text, telephone, or video consultation) by themselves.

...it is not necessarily the patients who should decide what needs to be done to investigate this properly. I have thought a lot about it, especially regarding these text-based consultations. It is imperative to stress that certain needs cannot be adequately met remotely and may require in-person attention for optimal outcomes.

ID5

Although citizens in general carry responsibility for contacting the health care system in a reasonable manner, the GPs feel personal responsibility for their listed patients. By directive, a Norwegian GP is obliged to respond to text consultations within 5 working days. But as previously noted, many of them feel drawn toward their inbox to prevent delays in urgent situations. Aware that several of their listed patients have limited health literacy, the GPs fear “user errors.” A feeling of moral duty to act may arise, even while being away from work.

Then the patient began sending text consultations expressing suicidal thoughts, and at that moment, I was not physically present. It was deeply concerning, prompting me to involve the entire medical center and dispatch the police to check on his well-being at his residence.

ID13

There was general concern among GPs that access to new digital services is likely to favor the digitally competent, leaving patients who require physical consultations behind on the priority list. Several participants associated the digital shift with the “Inverse Care Law” [51, 52] phenomenon, wondering who in society will benefit and who might be left behind in the digital future.

Initially, the respondents discussed that remote consultations will be more used by the young and healthiest who presumably are most digitally competent. However, further out in the discussions, the viewpoints became more nuanced. What one GP formulated as a future “digital care law” will not necessarily in general impede access for people with low education or socioeconomic status. According to many of the GPs’ experiences, most people from deprived social backgrounds have sufficient digital equipment and competence to book and perform remote consultations, and some even tend to do so frequently. What social deprivation entails, in this perspective, is a shortage of resourceful relational networks and practical health literacy. This contributes to limited ability to self-care and an inclination to access the health care system in an impulsive and inefficient manner. Although such patients may prefer simplified remote consultations, this preference may compromise long-term care quality.

The schedule is heavily burdened by tasks that don’t add value or patient care. How do we prioritize our time as a GP? [...] These patients steal a lot of my time...

ID14

Concurrently, as a consequence, GPs fear their attention may be diverted from proactive initiatives and essential care for patients with genuine health care needs and low digital proficiency—often stemming from cognitive issues, old age, language barriers, or other factors.

### Retaining Clinical Core Values in a Changing World

**Figure 1:**
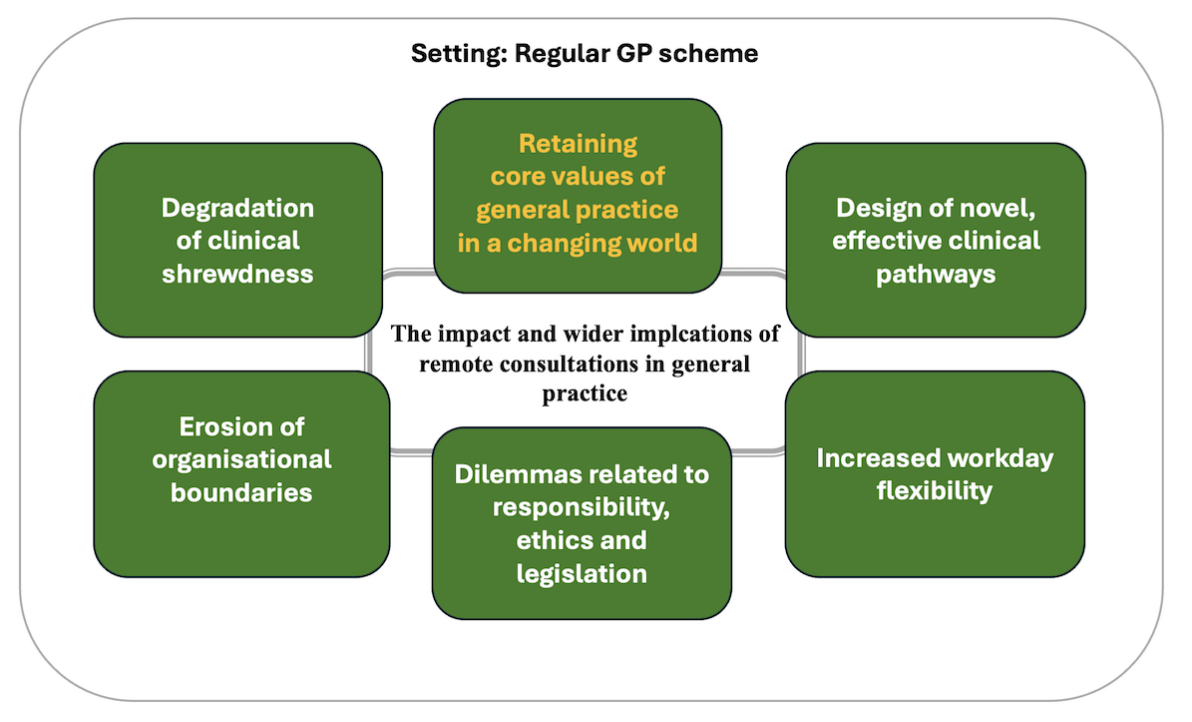
Main themes. GP: general practitioner.

This final theme emerged late in our analytical process. As depicted in Figure 1, we assign it a central, unifying position. All focus groups debated how remote consultations, for better or for worse, impact on the “new normal” state of general practice in overly complex ways. As said, they compared it with an ecosystem, where introduction of change in one arena (eg, the introduction of text consultations) appears to induce a range of consequences; direct and indirect, intended and nonintended, positive and negative. Ultimately, these changes are likely to affect care quality and patient safety, GPs’ time prioritizations, working style and well-being, job or home interface, and citizens’ access to equitable, high-quality health care. None of the focus groups came close to unequivocal conclusions regarding the overall impact. All groups, however, reached a consensus that there is no realistic or even desirable alternative to providing digital care in the context of the contract GP scheme.

If we do not do remote consultations, we will lose “the gold” in the contract GP scheme.

ID15

All groups, at some point, referred to the well-known metaphor among Norwegian contract GPs, namely “the gold of general practice.” Continuity of care is a central feature of this treasure. The GPs noted how consultations for relatively minor problems can contribute meaningfully to the establishment of a well-functioning doctor-patient relationship, as the doctor gets to know each individual patient’s general health status and ways of dealing with problems and challenges. In this context, the interviewed GPs expressed concern about being outmaneuvered by private health care providers operating outside the GP contract scheme, who claim to “ease the burden” on the system by managing the simplest cases on a large scale. The contract GPs hardly saw this as a threat to their personal business income (refer to explanation in Textbox 1). Rather, they viewed such all-private competitors as a threat to care quality, especially for inexperienced or mentally vulnerable patients in need of a stable and trustworthy contact and coordinator in the health care system. To avoid losing patients, the contract GPs discussed how they made compromises. Feeling obligated to meet societal and patient expectations, they sometimes gave in to “please the customer,” even if it meant conducting more remote consultations than they found clinically optimal.

We might gradually cede ground to alternative healthcare providers. I believe this would be unfortunate because, in terms of the quality of remote consultations, they heavily rely on the familiarity established through our longstanding patient-provider relationship, which is fundamental in our contract GP scheme.

ID11

Throughout the discussions, it was evident how the GPs were concerned with establishing “a new normal” with good equilibrium between physical and remote consultations. Facing this challenge, the GPs appeared open-minded and proactive, combining interest and optimism with high demands for critical reflection. The question is not whether, but how, to optimally implement remote consultations.

I'm adapting to my new way of working... What I find most challenging about remote communication methods is the multitude of possibilities they present, which I am determined not to overlook. On the contrary, I thoroughly enjoy engaging in them...

ID12

## Discussion

### Principal Findings

This qualitative focus group study provides insights into how remote consultations impact on contracted GP practices in Norway and how GPs perceive the implications of this uptake for the overall health care system and the core values of the discipline. On the positive side, remote consultations empower GPs to tailor effective clinical trajectories, seamlessly blending modalities to address diverse needs across clinical episodes—from initial triage to case closure. Remote consultations also introduce a welcome variability in daily routines. However, ease of access may unintentionally reduce patients’ tolerance for minor ailments and self-care. It may also compromise GPs’ ability to effectively detect and prioritize illness cases in genuine need of professional evaluation, that is, impede effective gatekeeping and equitable delivery of health care. Dilemmas arising at the intersection between GPs’ formally regulated responsibilities and self-perceived ethical obligations in the context of remote consultations contribute to increased work stress.

### Comparison With Previous Work

#### Reflecting on the Findings in Light of Existing Literature

Our study’s results align with Greenhalgh and colleagues’ [5] micro, meso, and macro-level analysis of remote consultations. These levels help us observe, assess, and explore new aspects within this framework.

#### Optimizing Episodes of Health Care by Integrating Remote Consultations

While many previous studies have addressed remote consultations as triage tools [36,38,39] or isolated communicative events [6-10,24], our study underscores the importance of optimizing their use according to their specific roles and functions within a clinical trajectory (at a meso level)—in line with World Health Organization’s recommendations [61]. Departing from the “episode” concept which describes a series of linked clinical contacts [16], we document how a contemporary episode can play out on diverse platforms, leveraging the unique strengths and weaknesses of different consultation modalities. Previous literature on the role of remote consultations in clinical pathway design is limited. A study from secondary health services emphasizes a general potential for efficiency [62], and another discusses the role of the GP and the operationalization of email consultations [28]. We provide in this study novel perspectives on medical trajectory design, in the context of the contract GP scheme, with increasing awareness among GPs when and how remote consultations should be used. Our findings point to some specific potentials for improved GP-patient proximity and continuity of care. We note how remote consultations facilitate what we call the “effortless commencement phenomenon” for semiacute cases and “just-in-time consultations” with unstable patients in addition to the more self-evident gain of maintaining contact despite geographical distance. We believe that such aspects—pertaining to continuity of care, clinical problem-solving, and GP-patient relationships—deserve further exploration [44]. GPs and patients across different countries may benefit from guidance from authorities regarding the desirable use of remote consultations.

An interesting finding in our study pertains to ergonomic advantages. It shows how GPs can benefit from strategic alternation between different consultation modalities during the workday based on their personal assessment of each modality’s intensity and requirements, combined with individual patient characteristics. Our findings are supported by previous studies that have concentrated on alleviating daily pressures [24,25]. However, the new-found flexibility also has its downsides at the level of work-related health. While facilitating working from home, when necessary, it may also intrude on doctors’ ability to relax and recover.

#### The Strained Gatekeeper

A fundamentally important question related to the overall impact of remote consultations pertains to quality. From previous research, it is well-documented that increased workload correlates with reduced self-care among GPs [6,24,63,64], including cognitive effects. This pressure may compromise the GP gatekeeper function [65,66], potentially increasing the burden on secondary health services [65]. In the context of assessment quality, there is ongoing debate about whether remote consultations serve as substitutes for, complements to, or precede traditional in-person consultations [22,24,34]. Our findings suggest that the rise in digital care, particularly text consultations, appears to reduce patients’ tendency to wait before seeking medical attention. In addition, text consultations seem to be carried out increasingly in addition to in-person consultations. Here there is room for more in-depth exploration.

All the focus groups delved further on the perceived decline in self-care capability among patients and a decreased threshold for seeking medical attention. Consistent with Barsky’s [64] assertions in the seminal 1988 paper “The paradox of health,” the participants of our study thought the digital transition in the middle of a pandemic had accelerated a societal trend toward heightened awareness of bodily symptoms and alarm regarding illness. Limited research explores how increased contacts and decreased disease incidence impact GPs’ judgment and gatekeeping quality. However, some studies suggest the deterioration of clinical skills as a result of reduced hands-on experience [5,34,54,63]. Our findings highlight challenges in identifying serious illnesses from sequences of digital inquiries evaluated under time pressure. Clinical reasoning and responsible risk management are context-dependent [67,68], and remote consulting as such has been shown to increase cognitive demands [69]. So-called clinical intuition (“gut feeling”) can be valuable in diagnostics [70]. Our findings align with previous research on the fallacies of clinical interpretations as a consequence of changed expectations [71,72]. Deliberate training and optimal working conditions appear important to combat clinical bluntness and safeguard quality.

#### The Digital Care Law Revisited

As already noted, previous studies on remote consultations have evoked Hart’s seminal paper “The Inverse Care Law” from 1971 [51], extending it to a “Digital Inverse Care Law” [52,73]. Existing research primarily concentrates on digital exclusion [74,75]; however, a recent paper by Dakin and colleagues [76] explores the phenomenon of patients creating insufficient digital presentations: These may lead to misdirection or deprioritization in remote consultation triage. Thereby, Dakin’s paper validates our finding that in relatively affluent societies where most people are literate and have access to digital tools, more subtle processes than simple digital exclusion should be anticipated in relation to inequities in digital access to quality care. From this macro viewpoint, our GPs expressed apprehension that social deprivation associated with low health literacy could lead to uncritical use of remote consultations, resulting in frequent health care contacts, albeit with suboptimal quality. Concomitantly and indirectly, other patients’ access to care is hampered. Faced with a flow of incoming requests, the GP’s attention may furthermore be diverted away from proactive care for patients with significant medical needs who hesitate or struggle to access the system [1,6,63]. Together, these dilemmas add to an emerging list of potentially overlooked ethical dimensions associated with remote consultations [26].

Although our study did not specifically aim to decipher the pros and cons of different consultation modalities, it highlights important context-dependent phenomena, for instance how text consultations allow patients direct access to their GPs without triage, in contrast to the booking systems for telephone or video consultations. This practice places additional strain on GPs and mirrors issues seen in the early use of telephone consultations [24,37]. The critical role of health secretaries in triage and counseling in primary care is likely underrecognized and inadequately documented [75]. We advocate for a strategic approach to triage of remote consultations to better identify patients most in need of medical attention [77,78].

A striking finding among the interviewed GPs is how they collectively strive to uphold the established core values of general practice [65,79]. Their willingness to engage in more remote consultations extends beyond the point that they deem professionally optimal—aiming to maintain continuity of care. The potential for excessive use must be carefully considered, balancing GPs’ management of clinical uncertainty with their commitment to equitable health services [5,55,78-80]. This is particularly crucial as patients may opt for care from other health care providers lacking continuity. Further ethical dilemmas regarding GPs’ legal responsibilities and ethical obligations in remote consultations complicate the situation in the strive for a new health care equilibrium.

### Strengths And Limitations

A strength of this study lies in the successful recruitment of experienced GPs with diverse characteristics, including variations in gender, age, remuneration systems, and geographical backgrounds. While 2 of the 5 focus groups were conducted digitally, these sessions still yielded high-quality data, suggesting that the remote format was well-suited for our purpose. Furthermore, we note how our participants’ substantial clinical experience allowed them to decipher and articulate quite clearly the impact of the digital shift on their individual practices. While including GPs earlier in their careers might have introduced a broader range of perspectives on the here-and-now situation, it would not necessarily have increased our study’s information power regarding the impact of the digital shift, as outlined in the literature [81,82].

Third, the study was conducted in the period following the pandemic, after restrictions had been lifted. However, we cannot claim that a fully stable “new normal” has been established. For example, GPs were still receiving full remuneration for telephone consultations, comparable with video and text consultations, which may have influenced their continued use. Despite this, our participants engaged actively with the new modalities (video and text), leading to insightful discussions. Thus, while the situation may not fully represent a long-term norm, the study effectively captures a significant impact of the digital transition on the practice and services of contract GPs.

Finally, although our study is based on a GP contract scheme, we believe our findings have relevance to other health care systems with varying funding models and regulations. As a model for broader application, the study demonstrates how remote consultations can have far-reaching effects on a health care system as a whole. It highlights the necessity of closely monitoring these effects, both in terms of clinical services and clinicians’ daily practices. Such monitoring can evidently be strengthened by integrating direct patient or user perspectives.

### Conclusion and Directions for the Future

We conclude that the widespread adoption of remote consultations in the Norwegian contract GP scheme is fundamentally reshaping the dynamics of GP work and the overall health care system. Contract GPs in Norway are working to balance the pros and cons of remote consultations, aiming to integrate them smoothly and designing novel medical trajectories. While remote consultations offer flexibility in daily routines, they may inadvertently reduce patients’ tolerance for minor ailments and self-care, potentially affecting GPs’ ability to prioritize serious cases. The consultation modality should be selected on the basis of perceived medical need and capacity rather than direct patient demand. Awareness and proactive management of the changes of digitalization are essential for maintaining sustainable, high-quality primary health care in accordance with the discipline’s core values. The main conclusion is that continuity in the doctor-patient relationship enhances the effectiveness of remote consultations and supports their broader integration into general practice.

## Data Availability

The datasets generated and analyzed in this study are securely housed within approved NTNU Faculty repositories. Although they are not publicly accessible, requests for access to anonymized data will be considered on a case-by-case basis. The limitations on public data sharing are in accordance with Norwegian legislation governing data protection and privacy.

## Appendix

Multimedia Appendix 1 Supplementary Material 1: Interview guide Supplementary Material 2: Information and consent scheme / Application for privacy and anonymity Table S1. Consolidated criteria for reporting qualitative studies (COREQ): 32-item checklist Table S2. Characteristics of participating GPs. Thick lines separate the five different focus groups.

## Abbreviations

COREQ: consolidated criteria for reporting qualitative research
GP: general practitioner
