# Supplementary material for: The Impact and Wider Implications of Remote Consultations for General Practice in Norway: Qualitative Study Among Norwegian Contract General Practitioners
Source: JMIR Form Res. 2024 Dec 17;8:e63068. doi: 10.2196/63068 (PMC11688597; doi:10.2196/63068)
Supplement: Multimedia Appendix 1 [file formative_v8i1e63068_app1.docx]

# Appendix

### Supplementary Material 1

###

### Interview guide

### *(Translated from Norwegian)*

### "Welcome and thank you for participating in this focus group. Our study aims to evaluate remote health services with general practitioners (GPs). Your participation is entirely voluntary, and you may withdraw at any time without any consequences.

### We will be recording today's session to accurately capture your insights. Rest assured that your identity will remain anonymous in any reports or publications resulting from this research. All data will be securely stored and kept confidential.

### We may ask you, or you may ask us, to review our notes or interpretations of today's discussion to ensure they accurately reflect your views. We have previously conducted other studies in the field, which has helped us ensure the reliability of our research and develop this interview guide.

### Please respect each other's perspectives during the discussion and feel free to speak openly. Your insights are invaluable to us. Do not be afraid to express your honest opinions and relate them to your experiences. Different viewpoints enrich the research, making it deeper and more valuable. Thank you again for your participation. Let us begin.

### Demographic info, “warm-up questions.”

### What proportion would you estimate digital consultations (text/telephone/video) make up in your practice?

### How does digital *communication affect your everyday working life?*

### Now we have been through a longer pandemic and digitization - Do you see the contours of a new "optimal" way of working?

### Do digital consultations *replace* the physical ones, or do they come in addition?

### Are we losing some of the *"gold"* in general medicine? Is something “human” being lost?

### What is the best/worst things about text consultations?

### What is the best/worst things about video consultations?

### What is the best/worst things about telephone consultations?

### When is it appropriate to utilize digital consultations? For which patients, situations, or types of problems are they most suitable?

### We have heard doctors say that video or text can be a great way to get to know new patients...?

### What communication techniques are employed for video, text, and telephone consultations?

### How is relationship-building facilitated during digital consultations?

###

### *(In Norwegian)*

### Norsk Intervjuguide

### Velkommen og takk for at du deltar i denne fokus-gruppen! Denne studien tar sikte på å evaluere digitale helsetjenester (tekst/telefon/video) med fastleger. Din deltakelse er helt frivillig, og du kan trekke deg når som helst uten at det får konsekvenser.

### Vi vil gjøre opptak av dagens sesjon for å kunne studere bidragene i etterkant. Alle data vil lagres sikkert og holdes konfidensielt. Du kan også være trygg på at identiteten din vil forbli anonym i alle rapporter eller publikasjoner som er et resultat av denne forskningen. Vi kan be deg, eller du kan be oss om å gjennomgå notatene eller tolkningene våre av dagens diskusjon for å sikre at de gjenspeiler dine synspunkter nøyaktig. Vi har tidligere utført andre studier innen feltet, noe som har hjulpet oss med å sikre påliteligheten til forskningen vår og utvikle denne intervjuguiden.

### Vennligst respekter hverandres synspunkter og perspektiver under diskusjonen, og snakk gjerne åpent. Din innsikt er verdifull for oss! Ikke vær redd for å uttrykke dine ærlige meninger og relatere dem til dine erfaringer. *Ulike* synspunkter beriker forskningen, og gjør den dypere og mer verdifull. Takk igjen for din deltakelse! La oss begynne.

### Demografisk informasjon og oppvarmingsspørsmål.

### Hvor stor andel vil du anslå at digitale konsultasjoner (tekst/telefon/video) utgjør i praksisen din?

### Hvordan påvirker digital kommunikasjon arbeidshverdagen din?

### Nå har vi vært gjennom en lengre pandemi og digitalisering – Ser du konturene av en ny «optimal» måte å jobbe på?

### Erstatter digitale konsultasjoner de fysiske, eller kommer de i tillegg?

### Mister vi noe av «gullet» i allmennmedisin? Mister vi noe mellommenneskelig?

### Hva er det beste/verste med tekstkonsultasjoner?

### Hva er det beste/verste med videokonsultasjoner?

### Hva er det beste/verste med telefonkonsultasjoner?

### Når er det hensiktsmessig å bruke digitale konsultasjoner – for hvilke pasienter, situasjoner og type problemer passer det best?

### Vi har hørt leger si at video eller tekst kan være en fin måte å bli kjent med nye pasienter på...?

### Hvilke kommunikasjonsteknikker bruker du i video/tekst/telefonkonsultasjoner?

### Hvordan skjer relasjonsbygging under digitale konsultasjoner?

###

###

###

## Supplementary Material 2

###

### Information and consent scheme / Application for privacy and anonymity

### (same text used for both purposes)

###

###

### *(Translated from Norwegian)*

### Regarding Participation in the Research Project:

### Evaluation of Remote Health Services with General Practitioners

###

### *Dear Doctor,*

### This is an invitation to you to participate in a 1.5-2-hour FOCUS group research project aimed at evaluating remote health services with general practitioners.

### In this letter, we provide information about the project's goals and what participation will entail for you.

### Purpose

### The project's purpose is to respond to the authorities' inquiries on how remote consultations affect the daily lives of general practitioners (GPs) and their impact on patients and society. The project is initiated and supported by the Norwegian Research Council (NFR). We will evaluate the new daily routines of GPs concerning remote health services, including video consultations, text-based consultations, and the use of phone consultations.

### We include a preparation/homework assignment before the focus group interview, where you are asked to think about a time when a video consultation worked particularly well and a time when it did not. We want to explore how video consultations and other remote consultation forms affect your daily life. The focus group questions can be sent to you if you wish to reflect beforehand.

### This is a research project and part of a doctoral study for Eli Kristiansen and Børge L. Norberg in Trondheim. The interviews will be audio-recorded and transcribed within a month of the interview date, after which all audio recordings will be deleted. All participant names and any identifiable situations will be anonymized or removed.

### The results of these interviews will be used to formulate questions for a larger evaluation of digital consultations through SKIL in Bergen, where video consultations will play a major role. This will be conducted later.

###

### Who is Responsible for the Research Project?

### NTNU and the National Center for eHealth Research (NSE) are responsible for the project. The Norwegian Research Council (NFR) is the client.

###

### Why Are You Being Asked to Participate?

### The focus groups will include GPs that are dedicated district-GPs with a common connection to the National Center for Rural Medicine led by Anette Fosse, or dedicated GPs in influential educational groups from all around Norway, and some purposely selected GPs with or without connection to the medical curriculum course at NTNU.

### You are being asked to participate because you are considered capable of reflecting on and elaborating how video consultations and other digital communication forms affect the daily lives of GPs in Norway.

###

### What Does Participation Involve?

### If you choose to participate in the project, you will take part in a focus group among colleagues in a secure setting. This will take approximately 1.5 hours.

###

### Voluntary Participation

### Participation in the project is voluntary. If you choose to participate, you can withdraw your consent at any time without giving a reason. All your personal data will then be deleted. There will be no negative consequences for you if you decide not to participate or later choose to withdraw.

###

### Your Privacy – How We Store and Use Your Information

### We will only use your information for the purposes described in this document. We will treat your information confidentially and in accordance with privacy regulations. Only the research team will have access to the audio files before they are transcribed. Your name and contact information will be kept confidential. Data from the audio files will be stored on a research server, locked, and encrypted before being permanently deleted.

### None of the participants will be identifiable in any publications, and no identifiable information from you or your examples will be published.

###

### What Happens to Your Information After the Research Project Ends?

### All information will be anonymized within a month (as mentioned earlier) and deleted when the project is completed/the thesis is approved, which is planned for the end of 2025.

###

### Your Rights

### As long as you can be identified in the data material, you have the right to:

### Access the personal data registered about you and receive a copy of the information,

### Correct personal data about you,

### Delete personal data about you, and

### Submit a complaint to the Data Protection Authority regarding the processing of your personal data.

###

### What Gives Us the Right to Process Your Personal Data?

### We process your data based on your consent.

### On behalf of NTNU and NSE; NSD (Norwegian Centre for Research Data AS) has assessed that the processing of personal data in this project complies with privacy regulations.

###

### Where Can I Find Out More?

### If you have questions about the study or wish to exercise your rights, contact: Børge Lønnebakke Norberg at NSE and NTNU, telephone number 91111814.

### If you have questions regarding NSD’s assessment of the project, you can contact: NSD – Norwegian Centre for Research Data AS by email ([personverntjenester@nsd.no](mailto:personverntjenester@nsd.no)) or by phone: 55 58 21 17.

###

### *Best regards,*

### *Børge Lønnebakke Norberg, Bjarne Austad (main supervisor), and Linn Getz (Professor, NTNU)*

###
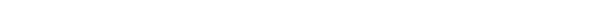


###

## Consent Form

### I have received and understood the information about the project “Evaluation of Remote Health Services with General Practitioners” and have had the opportunity to ask questions. I consent to:

### Participating in the focus group interview

### My data being processed until the project is completed”

###

### *(Signed by project participant, date)*

###

###

###

### *(Original Norwegian)*

### Om deltakelse i forskningsprosjektet:

### Evaluering av digitale helsetjenester hos fastlegen

### Dette er til deg som skal delta i 1,5 times FOKUS-gruppe forskningsprosjektet - hvor formålet er å evaluere digitale helsetjenester hos fastlegen.

###

### I dette skrivet gir vi deg informasjon om målene for prosjektet og hva deltakelse vil innebære for deg.

###

### Formål

### Formålet med prosjektet er å besvare myndighetenes ønske om hvordan elektroniske konsultasjoner påvirker hverdagen for fastleger, men også hvordan dette påvirker pasienter og samfunnet som helhet. Prosjektet er initiert og støttet av NFR (Norges Forskningsråd). Vi skal evaluere fastlegens nye arbeidshverdag vedrørende digitale helsetjenester som video-konsultasjoner, tekstbaserte konsultasjoner og bruk av telefon.

###

### Vi legger ved en forberedelse / hjemmelekse før fokusgruppe-intervjuet, der du skal tenke over ett tilfelle hvor video-konsultasjon fungerte *spesielt* bra - og en gang det ikke fungerte så godt. Vi vil utforske hvordan video-konsultasjoner og andre digitale konsultasjonsformer påvirker hverdagen din. FOKUS-gruppe-spørsmålene kan sendes til deg, dersom du ønsker å reflektere på forhånd.

###

### Dette er et forskningsprosjekt og samtidig en del av en doktorgrads-studie for Eli Kristiansen i Tromsø og Børge L Norberg i Trondheim. Intervjuene blir tapet på lydbånd og transkribert innen en måned fra intervjuets dato, og etter dette slettes alle lydspor fra intervjuene. Alle navn på deltakere og eventuelle gjenkjennbare situasjoner gjøres *u-gjenkjennbare* eller *slettes*.

###

### Resultater av disse intervjuene skal brukes til å utforme spørsmål i en større evaluering av digitale konsultasjoner gjennom SKIL i Bergen, som skal gjennomføres et senere tidspunkt, sannsynligvis i 2023.

###

### Hvem er ansvarlig for forskningsprosjektet?

### **NTNU og NSE (Nasjonalt senter for e-helseforskning)** er ansvarlig for prosjektet. NFR er oppdragsgiver.

###

### Hvorfor får du spørsmål om å delta?

### Utvalget er trukket ut blant leger som benytter seg av kvalitets-portalen SKIL i Bergen, er ansatte ved LPK-kurset ved NTNU eller er funnet blant dedikerte distriktsleger, ofte med et felles multiplum hos Nasjonalt senter for Distriktsmedisin ved leder Anette Fosse.

###

### Du blir spurt om å delta fordi du på en eller annen måte er vurdert å kunne reflektere og utdype hvordan video-konsultasjoner og andre digitale kommunikasjonsformer påvirker hverdagen for fastlegene i Norge. Kontaktopplysninger om deg ligger kun hos de respektive sentra.

###

### Hva innebærer det for deg å delta?

### **Hvis du velger å delta i prosjektet, innebærer det at du deltar i en FOKUS-gruppe blant kolleger i en trygg ramme. Det vil ta deg ca. 1,5 timer.**

###

### Det er frivillig å delta

### Det er frivillig å delta i prosjektet. Hvis du velger å delta, kan du når som helst trekke samtykket tilbake uten å oppgi noen grunn. Alle dine personopplysninger vil da bli slettet. Det vil ikke ha noen negative konsekvenser for deg hvis du ikke vil delta eller senere velger å trekke deg.

###

### Ditt personvern – hvordan vi oppbevarer og bruker dine opplysninger

### Vi vil bare bruke opplysningene om deg til formålene vi har fortalt om i dette skrivet. Vi behandler opplysningene konfidensielt og i samsvar med personvernregelverket. Kun forskerteamet vil ha tilgang til lydfilene før de transkriberes. Navnet og kontaktopplysningene dine vil. Datamaterialet fra lydfilene vil lagres på forskningsserver, innelåst og kryptert, før de slettes for godt.

###

### Ingen av deltakerne vil kunne gjenkjennes ved publisering, og ingen gjenkjennbare opplysninger fra hverken deg eller dine kasuistikker vil publiseres.

###

### Hva skjer med opplysningene dine når vi avslutter forskningsprosjektet?

### Alle opplysningene anonymiseres innen en måned (se tidligere) og slettes når prosjektet avsluttes/oppgaven er godkjent, som etter planen er ved utgangen av 2025, men kan bli forlenget.

###

### Dine rettigheter

### Så lenge du kan identifiseres i datamaterialet, har du rett til:

### innsyn i hvilke personopplysninger som er registrert om deg, og å få utlevert en kopi av opplysningene,

### å få rettet personopplysninger om deg,

### å få slettet personopplysninger om deg, og

### å sende klage til Datatilsynet om behandlingen av dine personopplysninger.

###

### Hva gir oss rett til å behandle personopplysninger om deg?

### Vi behandler opplysninger om deg basert på ditt samtykke.

###

### På oppdrag fra NTNU og NSE har NSD – Norsk senter for forskningsdata AS vurdert at behandlingen av personopplysninger i dette prosjektet er i samsvar med personvernregelverket.

###

### Hvor kan jeg finne ut mer?

### Hvis du har spørsmål til studien, eller ønsker å benytte deg av dine rettigheter, ta kontakt med: Børge Lønnebakke Norberg ved NSE og NTNU Ulf 91111814.

### Hvis du har spørsmål knyttet til NSD sin vurdering av prosjektet, kan du ta kontakt med:

### NSD – Norsk senter for forskningsdata AS på epost ([personverntjenester@nsd.no](mailto:personverntjenester@nsd.no)) eller på telefon: 55 58 21 17.

###

###

###

### *Med vennlig hilsen*

###

### Børge Lønnebakke Norberg, Bjarne Austad (hovedveileder) og Linn Getz (Professor NTNU)

###

###

###

###

###

### Samtykkeerklæring

###

### Jeg har mottatt og forstått informasjonen om prosjektet «Evaluering av digitale helsetjenester hos fastlegen», og har fått anledning til å stille spørsmål. Jeg samtykker til:

###

### å delta i FOKUS-gruppe-intervju

### Jeg samtykker til at mine opplysninger behandles frem til prosjektet er avsluttet

###

### --------------------------------------------------------------------------------------------------

### *(Signert av prosjektdeltaker, dato)*

# Table S1. Consolidated criteria for reporting qualitative studies (COREQ): 32-item checklist

| **Domain 2: study design** |  |  |
| --- | --- | --- |
| *Theoretical framework* |  |  |
| 9. Methodological orientation and Theory | What methodological orientation was stated to underpin the study? e.g. grounded theory, discourse analysis, ethnography, phenomenology, content analysis | 9 |
| *Participant selection* |  |  |
| 10. Sampling | How were participants selected? e.g. purposive, convenience, consecutive, snowball | 7 |
| 11. Method of approach | How were participants approached? e.g. face-to-face, telephone, mail, email | 8 |
| 12. Sample size | How many participants were in the study? | 7 |
| 13. Non-participation | How many people refused to participate or dropped out? Reasons? | 7 |
| *Setting* |  |  |
| 14. Setting of data collection | Where was the data collected? e.g. home, clinic, workplace | 8 |
| 15. Presence of non-participants | Was anyone else present besides the participants and researchers? | 8 |
| 16. Description of sample | What are the important characteristics of the sample? e.g. demographic data, date | 7-8 |
| *Data collection* |  |  |
| 17. Interview guide | Were questions, prompts, guides provided by the authors? Was it pilot tested? | 8 |
| 18. Repeat interviews | Were repeat inter views carried out? If yes, how many? | 8 |
| 19. Audio/visual recording | Did the research use audio or visual recording to collect the data? | 8 |
| 20. Field notes | Were ﬁeld notes made during and/or after the interview or focus group? | 8 |
| 21. Duration | What was the duration of the inter views or focus group? | 9 |
| 22. Data saturation | Was data saturation discussed? | 8,9,26 (incl. information power) |
| 23. Transcripts returned | Were transcripts returned to participants for comment and/or correction? | - |
| **Domain 3: analysis and ﬁndings** |  |  |
| *Data analysis* |  |  |
| 24. Number of data coders | How many data coders coded the data? | 8 |
| 25. Description of the coding tree | Did authors provide a description of the coding tree? | 10 (fig.) |
| 26. Derivation of themes | Were themes identiﬁed in advance or derived from the data? | Of Course, from |
| 27. Software | What software, if applicable, was used to manage the data? | 9 |
| 28. Participant checking | Did participants provide feedback on the ﬁndings? | Yes (not mentioned) |
| *Reporting* |  |  |
| 29. Quotations presented | Were participant quotations presented to illustrate the themes/ﬁndings? Was each quotation identiﬁed? e.g. participant number | Yes, 9 |
| 30. Data and ﬁndings consistent | Was there consistency between the data presented and the ﬁndings? | 9 |
| 31. Clarity of major themes | Were major themes clearly presented in the ﬁndings? | 9 |
| 32. Clarity of minor themes | Is there a description of diverse cases or discussion of minor themes? | 22; themes were developed to a higher level according to B&C |

# Table S2. Characteristics of participating GPs. Thick lines separate the five different focus groups.

| **No.** | **Age category**  **(yrs.)** | **Sex** | **Use of video, telephone or text consultations** | **Specialist in general practice** | **Geographical location** | **List size**  **(approx.)** | **Interview format** |
| --- | --- | --- | --- | --- | --- | --- | --- |
| 1 | 30-40 | F | Video, text, telephone | Under specialization | Rural | 500 | Digital |
| 2 | 30-40 | M | Video, text, telephone | Yes | Rural | 1000 | Digital |
| 3 | 40-50 | F | Video, text, telephone | Yes | Rural | 500 | Digital |
| 4 | 50-60 | F | Video, text, telephone | Yes | Rural | 600 | Digital |
| 5 | 50-60 | F | Video, text, telephone | Yes | Urban | 1100 | Digital |
| 6 | 50-60 | F | Video, text, telephone | Yes | Urban | 1300 | Digital |
| 7 | 40-50 | F | Video, text, telephone | Yes | Urban | 1200 | Digital |
| 8 | 50-60 | F | Video and telephone  Text turned off because of high demand | Yes | Urban | 1200 | Digital |
| 9 | 40-50 | M | Video, text, telephone | Yes | Urban | 1000 | Physical |
| 10 | 60-70 | M | Video, text, telephone | Yes | Urban | 900 | Physical |
| 11 | 40-50 | F | Video, text, telephone | Yes | Urban | 800 | Physical |
| 12 | 40-50 | F | Video, text, telephone.  Limits access to text because of high demand | Yes | Rural-urban fringe | 900 | Physical |
| 13 | 40-50 | M | Video, text, telephone | Yes | Rural-urban fringe | 1200 | Physical |
| 14 | 40-50 | F | Video, text, telephone | Yes | Rural-urban fringe | 900 | Physical |
| 15 | 40-50 | M | Video, text, telephone | Yes | Urban | 1500 | Physical |
| 16 | 50-60 | M | Video, text, telephone | Yes | Some Urbanity | 1300 | Physical |
| 17 | 60-70 | M | Video, text, telephone.  Limits access to text because of high demand | Yes | Rural-urban fringe | 1200 | Physical |
| 18 | 30-40 | M | Video, text, telephone.  Limits access to text because of high demand | Yes | Rural-urban fringe | 800 | Physical |
